# Framing cognitive offloading in terms of gains or losses: achieving a more optimal use of reminders

**DOI:** 10.1186/s41235-022-00416-3

**Published:** 2022-07-16

**Authors:** Lea Fröscher, Ann-Kathrin Friedrich, Max Berentelg, Curtis Widmer, Sam J. Gilbert, Frank Papenmeier

**Affiliations:** 1 Department of Psychology, University of Tübingen, Tübingen, Germany; 2 Institute of Cognitive Neuroscience, University College London, London, UK

**Keywords:** Framing, Offloading, Reminders, Metacognition, Prospective memory

## Abstract

Nowadays individuals can readily set reminders to offload intentions onto external resources, such as smartphone alerts, rather than using internal memory. Individuals tend to be biased, setting more reminders than would be optimal. We address the question whether the reminder bias depends on offloading scenarios being framed as either gains or losses, both between-participants (Experiment 1) and within-participants (Experiment 2). In both experiments, framing of reminders in terms of gains resulted in participants employing a risk-averse strategy and using more reminders than would be optimal. Importantly, however, participants used reminders more optimally and were more willing to choose the risk-seeking option of remembering internally when reminders implied a loss. Based on metacognitive measures in Experiment 2, the reminder bias increased the more underconfident participants were about their memory abilities in both framing scenarios. Framing did not alter this relationship between erroneous metacognitive underconfidence and reminder bias but provides an additional influence. We conclude that emphasizing the losses (costs) associated with external reminders helps in achieving more optimal decisions in offloading situations, and that in addition to cognitive effort and metacognitive judgments, framing needs to be considered in improving individuals’ offloading behavior.

## Public significance statement

Faced with the choice of remembering with internal memory or using external reminders (e.g., smartphone apps, calendars), individuals generally tend to use reminders more than optimal. Our results suggest that emphasizing either the gains (benefits) or losses (costs) associated with external reminders influences individuals’ decision between using internal memory or external reminders. Emphasizing the losses (costs) associated with external reminders led individuals to rely less on external reminders and thus to make more optimal use of the combination of their internal memory resources and the external aids. Therefore, our findings may help to optimize the use of memory aids, that is to compensate for memory limitations in everyday life whenever necessary but at the same time to not neglect internal cognitive resources.

## Introduction

Suppose you have to make an appointment for 4 pm tomorrow. Part of everyday life requires remembering delayed intentions that are fulfilled in the future and stored in prospective memory (Einstein & McDaniel, 1990). However, the capacity of prospective memory is limited (e.g., Cherry & LeCompte, 1999), leading to frequent failures in remembering delayed intentions, which can interfere with functioning in everyday life (Boag et al., 2019; Ellis et al., 1999; Kliegel et al., 2000). As technology has become commonplace nowadays (e.g., smartphones or digital watches), individuals usually have the option to offload information onto external devices to help them remember intentions. Using external artifacts to reduce cognitive demand is known as cognitive offloading (Risko & Gilbert, 2016). For example, users can readily set up reminders on their smartphones to remind them of their appointment.

Multiple views have been proposed attempting to explain individuals’ choice between relying either on internal cognitive resources or external aids when solving tasks. A minimal memory view proposes that humans have the tendency to store information externally whenever possible (Ballard et al., 1995). This is consistent with findings suggesting that individuals aim to avoid actions associated with cognitive demand (Kool et al., 2010). However, this would also imply that individuals should always use offloading tools regardless of other factors, which is clearly not the case. In contrast, individuals’ choice between either offloading or relying on internal resources is determined by multiple factors, such as memory capacity (Meyerhoff et al., 2021), memory load (Gilbert, 2015a), monetary reward (Sachdeva & Gilbert, 2020), metacognitive judgments regarding one’s own internal abilities (Boldt & Gilbert, 2019; Gilbert, 2015b; Risko & Gilbert, 2016; but see Grinschgl et al., 2021a), or the interaction and interface design of offloading tools (Grinschgl et al., 2020). Other influence factors on offloading are more generic, such as context (e.g., time frame, device) or personal preferences (e.g., personality, consequences of a missed appointment).

The decision to engage in offloading behavior is also affected by cost–benefit considerations (Gray et al., 2006). The most obvious benefit of using offloading tools rather than relying on internal resources is that with offloading remembering the offloaded information is nearly guaranteed (Risko & Gilbert, 2016). This accuracy-related benefit of offloading accounts not only for daily life, such as remembering the items to buy in the grocery with either a shopping list or internal memory. It also accounts for well-established offloading tasks, such as the intention offloading task (e.g., Gilbert et al., 2020) or the task of remembering information with the support of writing it down (e.g., Risko & Dunn, 2015). In addition, outsourcing cognitive demand onto external tools can sometimes be considered less effortful compared to remembering internally (Ballard et al., 1995; Sachdeva & Gilbert, 2020).

External reminders also incur costs, however. In everyday life, these costs include the time and effort of setting them up, and the interruptions they can cause. These costs may be individually minimal. But they would mount to an unacceptable level when applied to the multitude of intentions maintained over a typical day, including those that are trivial or highly practiced such as remembering to go to work, brush one’s teeth, eat, or sleep. An additional possible cost of external reminders is that they may prevent an opportunity to strengthen internal cognitive skills (though see Scarampi & Gilbert, 2020).

### Optimal use of offloading

Considering the decision between relying on offloading tools versus internal memory as a tradeoff between costs and benefits raises the question of whether there might be an optimal solution for this tradeoff. With *optimality*, we refer to a decision that perfectly balances the costs (e.g., time and effort related to offloading) and the benefits of offloading (e.g., reduced cognitive demand and increased accuracy). Accordingly, we refer to decisions as *optimal* if they maximize the benefits while minimizing the costs of offloading, that is, relying on offloading tools as often as necessary but as seldom as possible. In turn, we define *biases* as deviations from this normative decision-making model.

To quantify optimality and bias in the context of cognitive offloading research, Gilbert et al. (2020) introduced the so-called *optimal reminders task*. In this task, participants are instructed to drag circles to the bottom of a square box in ascending numerical order. Sometimes, *special circles* that were briefly filled with a different color appeared, and participants had to remember the delayed intention of dragging those circles to the correspondingly-colored border when it was their turn. In order for participants to fulfill these intentions, they introduced them with two strategies: relying on internal memory or setting external reminders. Whereas in some trials, participants were forced to use either internal memory or external reminders, other trials gave participants a free choice between scoring a maximum amount of points using memory or a lesser amount of points using reminders. The number of points gained when using reminders was manipulated across trials. Based on participants’ performance in the forced trials, Gilbert et al. (2020) calculated a normative optimal points value at which participants should switch from using reminders to using memory. Based on actual choice behavior, they evaluated participants’ bias. They observed that participants did not show optimal choices. Instead, participants chose reminders more than optimal, thus demonstrating a bias toward reminders––the *reminder bias* (Gilbert et al., 2020).

The size of this reminder bias for delayed intentions can be influenced by individuals’ metacognitive judgments regarding the subjective perception of their internal abilities (Boldt & Gilbert, 2019; Risko & Gilbert, 2016). Reminder use was predicted by individuals’ erroneous underconfidence in their memory abilities (Gilbert, 2015a; Gilbert et al., 2020). Specifically, the reminder bias was higher among those individuals who were underconfident about their own memory (Engeler & Gilbert, 2020). However, when Gilbert et al. (2020) corrected participants’ confidence via positive feedback, thus aiming at debiasing participant’s offloading choices by making them less underconfident, participants were less biased toward using reminders but still offloaded more than would be optimal. In a similar vein, providing participants with a financial incentive based on overall performance reduced the reminder bias, thus causing participants to use reminders more optimally (Sachdeva & Gilbert, 2020), likely because participants were willing to invest more cognitive effort. However, despite the reduction in the reminder bias, this was again not enough to debias participant’s offloading choices. Thus, if used as a tool to achieve a more optimal use of reminders, both approaches were only partially effective in reducing the reminder bias, suggesting that there must be further barriers that first have to be overcome in order for the reminder bias to be fully eliminated.

### Offloading vs. internal memory: decision under uncertainty

We take a novel perspective on individuals’ offloading decisions. Considering the typically achieved performance when using an offloading strategy compared to internal memory, it turns out that performance is usually very high or even near perfect with offloading compared to a much more unreliable performance when relying on the internal memory. Thus, when choosing between offloading or internal memory, individuals decide between achieving a relatively certain outcome when using the offloading strategy and an uncertain or risky outcome when using internal memory.

When making decisions under risk, individuals typically prefer certain over uncertain choices, also known as the *certainty effect* (Kahneman & Tversky, 1979). For example, individuals would prefer winning $3000 with certainty over winning $4000 with 80% probability, even though the expected outcome is higher in the second variant. Interestingly, this bias reverses when the choice options are presented as losses rather than gains, also known as the *reflection effect* (Kahneman & Tversky, 1979). For example, individuals would prefer losing $4000 with an 80% probability over losing $3000 with certainty, even though the expected loss is lower in the second variant. That is, switching from gains to losses also causes a shift from risk-averse choice strategies to risk-seeking choice strategies (e.g., Baucells & Villaís, 2010; Kahneman & Tversky, 1979; Mather et al., 2012). This shift in choice strategies occurs not only if there is an actual change in outcome (win money vs. loose money), but it occurs also if the same outcome is phrased to appear to involve either gains or losses, the so-called *framing effect* (e.g., Bless et al., 1998; Fagley, 1993; Highhouse & Paese, 1996; for a review, see Kühberger, 1998; Piñon & Gambara, 2005; Steiger & Kühberger, 2018; Tversky & Kahneman, 1981). For example, under a gain framing, individuals would prefer the risk-averse option of winning $300 with certainty over the risky option of winning either $1000 with 30% probability or $0 with 70% probability. If the same outcome (average profit of $300) is framed under losses, individuals show a shift to a risk-seeking strategy. That is, if one tells individuals that they have an initial $1000 and that they could choose between the option of losing $700 with certainty and the option of losing either $0 with 30% probability or $1000 with 70% probability, individuals would prefer the later (risky) option.

Applying those findings from the literature on decision-making (Kahneman & Tversky, 1979) to cognitive offloading makes evident that the previously reported reminder bias (Gilbert et al., 2020) could also be seen as individuals employing a risk-averse choice strategy. Within previous research on the reminder bias (Engeler & Gilbert, 2020; Gilbert et al., 2020; Sachdeva & Gilbert, 2020), the choice between using reminders or internal memory was framed in terms of gains, such as earning some points using reminders and earning a maximum reward using memory. Applying the certainty effect to cognitive offloading, one would thus expect that individuals prefer outcomes that are near-certain (using an offloading strategy) over outcomes that are more uncertain (using internal memory), thus resembling the reminder bias.

Given the idea that the choice between cognitive offloading and internal memory represents a decision under uncertainty, a switch from gain framing to loss framing should also cause a switch from risk-averse choice strategies to risk-seeking choice strategies within the context of cognitive offloading. Thus, with the aim of using the framing of reminders as a means of achieving a more optimal use of reminders, reversing the framing from gains to losses might reduce, eliminate, or even reverse the reminder bias. With the present research, we investigated whether this shift in the reminder bias could be observed.

## Experiment 1

Our first experiment used the optimal reminders task (Gilbert et al., 2020) and expanded it by implementing a between-participants manipulation regarding the framing of reminders. We framed reminders as gains, just as previous research, or as losses, which had not been done before. We had two key hypotheses. In the gain framing condition, we expected to replicate previous findings on cognitive offloading, thus showing a reminder bias (Gilbert et al., 2020). For the loss condition, we expected a reduction of the reminder bias. This experiment was preregistered; see https://osf.io/rcu8v.

### Method

#### Participants

We recruited the participants using the student mailing list of the University of Tübingen and online posts shared in non-commercial Facebook and WhatsApp groups. Following exclusions (see below), our sample consisted of 141 participants (94 reporting their gender as male, 32 as female, and 2 as other). Participants had a mean age of 26.30 years (*SD* = 8.90, *range* = 18–64). The experiment took approximately 45 min, for which participants received the opportunity to win one of six €10 gift cards from a local bookstore or train company. We conducted this experiment in accordance with the APA guidelines for research ethics, and participants provided informed consent before participating.

We performed a power analysis based on the results observed in the unadvised group of Experiment 2 in Gilbert et al. (2020), using the R package powerbydesign (Papenmeier, 2018). To achieve a power of 80% for the investigated interaction effect (assumption of reminder bias under gain framing and no bias under loss framing; see the script containing the power simulation for details: https://osf.io/xqt8j), we required a sample size of 136 participants. We stopped data collection after two weeks, with the study slightly overshooting the targeted sample size (*N* = 141, 9 excluded) at this point in time.

#### Optimal reminders task

We modified the optimal reminders task used by Gilbert et al. (2020). In this task, participants can choose (a) to remember intentions using internal memory, which leads to a maximum reward for each remembered item, or (b) they can set external reminders, which leads to a smaller reward that varies from trial to trial. This paradigm allowed us to examine not only the frequency of reminder-setting but also its optimality. For example, suppose an individual’s accuracy is 55% when using their own memory and 100% when using reminders. If they are given a choice between earning 10 points per item using their own memory or 5 points per item using reminders, it is optimal to use the internal memory strategy. But if they are offered 6 points per item when using reminders, it is optimal to select this strategy instead. By comparing participants’ choices with the optimal strategy, this paradigm can be used to calculate whether individuals are (a) biased toward using external reminders, (b) biased toward using or their own memory, or (c) optimally calibrated.

**Fig. 1:**
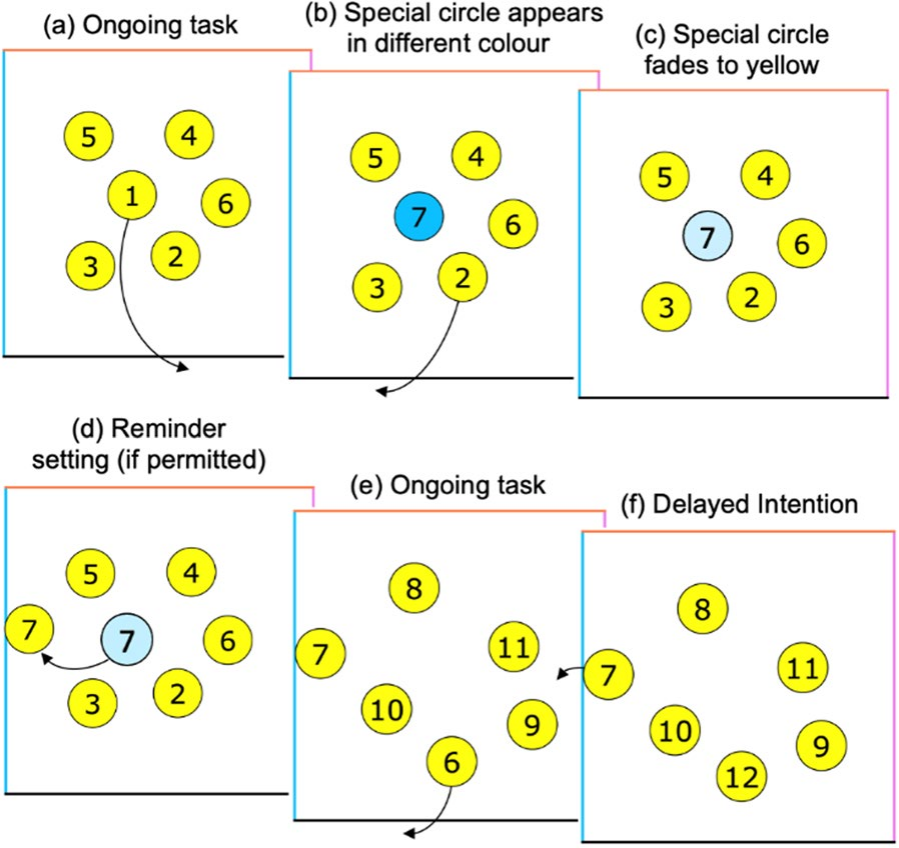
Illustration of the optimal reminders task. *Note* Example trial of the optimal reminders task. **a** Participants were instructed to drag circles to the bottom edge of a box in sequential order. Each time a circle was moved onto an edge, it disappeared from the screen and the next circle in sequence emerged; **b** Sometimes, new circles were initially highlighted in a different color, indicating a delayed intention to drag the special circle to the same-colored edge when reached in sequence; **c** A special circle’s color faded back to yellow two seconds after appearance. **d** If permitted, participants set reminders by instantly dragging the special circles near their intended edge when they emerged on the screen; **e** Participants carried on with the task of dragging circles to the bottom of the box; **f** After dragging the circles in sequential order, they could then execute the delayed intention to drag the special circle to its intended location

On each trial, participants used their computer mouse to sequentially drag 25 numbered circles to the bottom of a box (Fig. 1). Up to six circles were visible a time, and each time a circle was removed from the box, it was replaced with a new one (e.g., after dragging ‘1’ to the bottom, a new circle labeled ‘7’ appeared in its place). The left, top, and right edges of the box were colored blue, orange, and purple, respectively. Occasionally, new circles appeared initially in one of these colors before fading to yellow after 2 s. This was an instruction to form a delayed intention to drag these ‘special’ circles to the corresponding edge of the box. For example, if a special circle (e.g., 7) initially appeared as blue, participants needed to remember this instruction while they dragged circles 2 to 6 to the bottom of the box (by which time the special circle had faded to yellow). They could then execute the intention to drag 7 to the left. Within each trial consisting of 25 circles, 10 special circles were presented. These circles appeared between the 7th and 25th circle in the sequence. (The initial 6 circles were already on screen at the beginning of the trial, so they could not act as targets.) These 19 possible target positions were split into 10 adjacent bins (9 of which had a length of two and one of which, placed randomly in the sequence, had length one). One target was then placed randomly within each of these bins. As a result of the multiple concurrent intentions, participants were unlikely to remember all if they relied on internal memory alone. Alternatively, if they used reminders, they could offload the intentions by immediately dragging special circles next to the instructed edge when they first appeared (e.g., dragging a blue 7 toward the left edge of the box as soon as it appeared rather than waiting for it to fade to yellow first). The location of the special circle then acted as a reminder when the participant reached this number in the sequence. An everyday analogy would be leaving an object by the front door so that you remember it when leaving the house the next day.

**Fig. 2:**
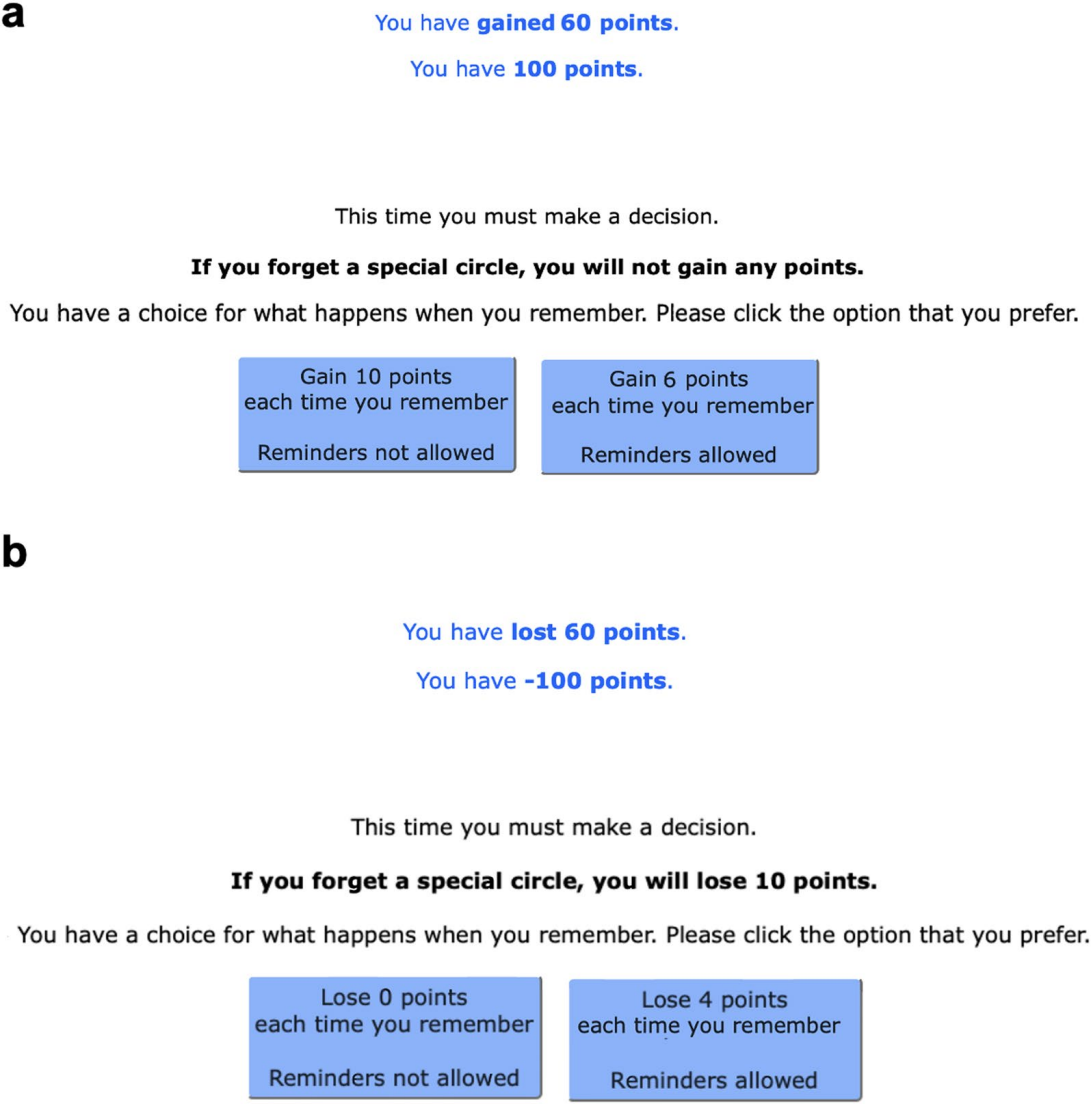
Example instructions for the free-choice trials. *Note* Prior to the start of each free-choice trial, participants were given the choice to either rely on their internal memory or to set external reminders. **a** In the gain condition, participants would score 10 points for each special circle they remembered if they relied on their memory or a smaller number of points (1–9) if they selected to set reminders. **b** In the loss condition, if participants relied on their memory, they would lose 0 points for each special circle they remembered. If they chose to use reminders, however, they would score minus points (− 9 to − 1). For both framing conditions, a sequence of 25 circles was presented in each trial, with 10 of them acting as special circles

Participants alternated between ‘forced’ and ‘choice’ trials. On forced trials, they had to use either their own memory (‘forced-internal’) or reminders (‘forced-external’). On choice trials, participants decided between earning 10 points per remembered item (using their own memory), or a smaller number of points between 1 and 9 (using external reminders). We calculated the optimal strategy based on performance on the forced trials, then compared this with their actual decisions on the choice trials. The experiment was split into two conditions (gain and loss), with participants being assigned randomly to one condition. In both conditions, participants were given 0 points at the beginning of the experiment. Further, participants in the gain condition chose between receiving 10 points for each remembered special circle or a smaller number of points (1–9) to use reminders (see Fig. 2). For each missed special circle, participants gained zero points. This matches the version of the task used in previous research (Gilbert et al., 2020). In the loss condition, participants lost 10 points for each missed special circle, and they had the choice between (a) using their own memory and keeping all their previously scored points (losing 0) each time they correctly remembered special circles or (b) using reminders and scoring minus points every time they remembered (− 9 to − 1). All instructions were presented in German.

#### Apparatus

Participants completed the task via their computer’s web browser. Participation was only permitted if the browser window had dimensions of at least 500 × 500 pixels. The square box containing the circles was sized at 80% of the horizontal or vertical extent of the browser window, whichever was smaller. Each circle had a radius of 5.5% of the width/height of the box, and all circles were initially placed so that they fall within a central portion of the box with dimensions sized at 56% of the total width/height, so that no circles were adjacent to any of the edges of the box at the beginning of the trial.

#### Procedure

After providing informed consent, the computer randomly assigned participants to one framing condition, with the assignment resulting in 63 and 78 participants for the gain and loss framing, respectively. Participants then completed the six practice trials: In the first trial, participants dragged the circles in sequence to the bottom of the square (8 circles in total). They further practiced by dragging one special circle to the instructed edge in the second practice trial (8 circles in total). They repeated this practice trial and were not allowed to continue the experiment until they responded correctly to this special circle. They then continued with two consecutive practice trials of actual length (10 special circles out of 25 circles in total). Following this, they were made aware of the ability to use reminders in this task. They practiced again, but on the forced-external trial type. This time, they needed to respond correctly to at least 8 out of 10 special circles in order to continue the experiment. In line with previous studies (Gilbert, 2015b; Gilbert, et al., 2020), we used the exact value of eight correct responses to ensure that participants were able to achieve at least 80% accuracy with using the external strategy. After performing one additional forced-external practice trial, participants were instructed about the upcoming forced and choice trials and that their task was to gain as many points as possible (lose as few points as possible).

During the main experiment, participants performed a total of 17 trials. On odd-numbered trials, participants were given a free choice between using internal memory (gain 10 points/lose 0 points; according to framing condition) or reminders (gain/lose 1–9 points per special circle, presented in random order). On even-numbered trials, participants alternated between the forced-external and forced-internal trials, with the starting trial type (external or internal) randomized between participants. The trial number was set to the exact number of 17 (9 free, 8 forced) trials to be consistent with previous versions of the optimal reminders task (Gilbert et al., 2020). After completion, participants were given the opportunity to enter the prize draw on SoSci Survey.

#### Reward

Participants were told that they were scoring points, with the prospect of earning (losing) up to 1700 points in the gain (loss) condition. Therefore, the earnings could range between 0 and 1700 points in the gain condition, and between minus 1700 points and 0 points in the loss condition. The experiment was promoted by offering participants the chance to win one of six 10€ gift cards for taking part in the experiment.

#### Design

The experiment employed a 2 (framing condition: gain vs. loss) $\times$ 2 (indifference point: optimal vs. actual) design with framing as a between-participants variable, and we defined five variables of interest:

- Forced-internal accuracy (ACC_FI_). This is the mean target accuracy (proportion of special circles correctly dragged to the instructed location) on forced-internal trials.
- Forced-external accuracy (ACC_FE_). This is the mean target accuracy (proportion of special circles correctly dragged to the instructed location) on forced-external trials.
- Optimal indifference point (OIP). For choice trials, this is the value for special circles offered with reminders at which an unbiased individual should be indifferent between the two options, based on the accuracy in the forced-internal and forced-external trials (ACC_FI_ and ACC_FE_). As in Gilbert et al. (2020), this was calculated as
  1 $${\text{OIP }} \times {\text{ACC}}_{{{\text{FE}}}} = 10 \times {\text{ACC}}_{{{\text{FI}}}}$$

If the OIP was less than 1 or greater than 9, it was set to the relevant lower or upper bound. This was so that the potential values of the OIP would match the potential values of the point at which they were actually indifferent, which was bound by their choices for values 1 to 9.

- Actual indifference point (AIP). This is the estimated point for choice trials at which participants were actually indifferent to the two strategy options. As in Gilbert et al. (2020), this was calculated by fitting a sigmoid curve to the strategy choices (0 = own memory; 1 = reminders) across the nine special values (1–9), using the quickpsy function from the R package quickpsy (Linares & López-Moliner, 2019) bounded to the range 1 to 9 (see the analysis script on OSF; https://osf.io/qsfmy/). Based on this curve, we were able to estimate the point associated with 50% probability of choosing either strategy, which is the AIP.
- Reminder bias. This is defined as OIP–AIP, which will yield a positive value for a participant biased toward using more reminders than would be optimal, and a negative value for a participant biased toward using fewer reminders than would be optimal.

Each of the previous five measures was calculated separately for the gain and loss condition, and to compare AIP and OIP between framing conditions, calculation of indifference points was performed after transforming the minus points from the loss framing condition by adding 10.

#### Exclusion criteria

In accordance with our preregistration, we excluded participants if (a) their accuracy in forced-internal trials (averaged across gain and loss conditions) was lower than 10% (*n* = 0); (b) accuracy in the forced-external trials was lower than 70% (averaged across gain and loss conditions; *n* = 0); (c) accuracy in the forced-internal trials was higher than in the forced-external trials in either condition (*n* = 0); (d) there was a negative point biserial correlation between points offered for correct responses on each trial using reminders (1–9) and choice of strategy (0 = own memory, 1 = reminders; this excluded participants who were more likely to set reminders when it earned them fewer points, indicating random strategy selection; *n* = 8); and (e) their reminder bias score exceeded 2.5 standard deviations from the group mean (*n* = 1). The data are publicly available (https://osf.io/8shkf/).

#### Transparency and openness

For the study’s entire research report, we communicate our methodical and statistical approach including sample size estimation, data exclusion, experimental manipulation, and measures of interest. All hypotheses, experimental methods, and planned analyses were preregistered before data collection. All analyses were run in R, and information on the R environment (including package versions) used for the analyses is given in the analysis script.

### Results

#### Accuracy

**Fig. 3:**
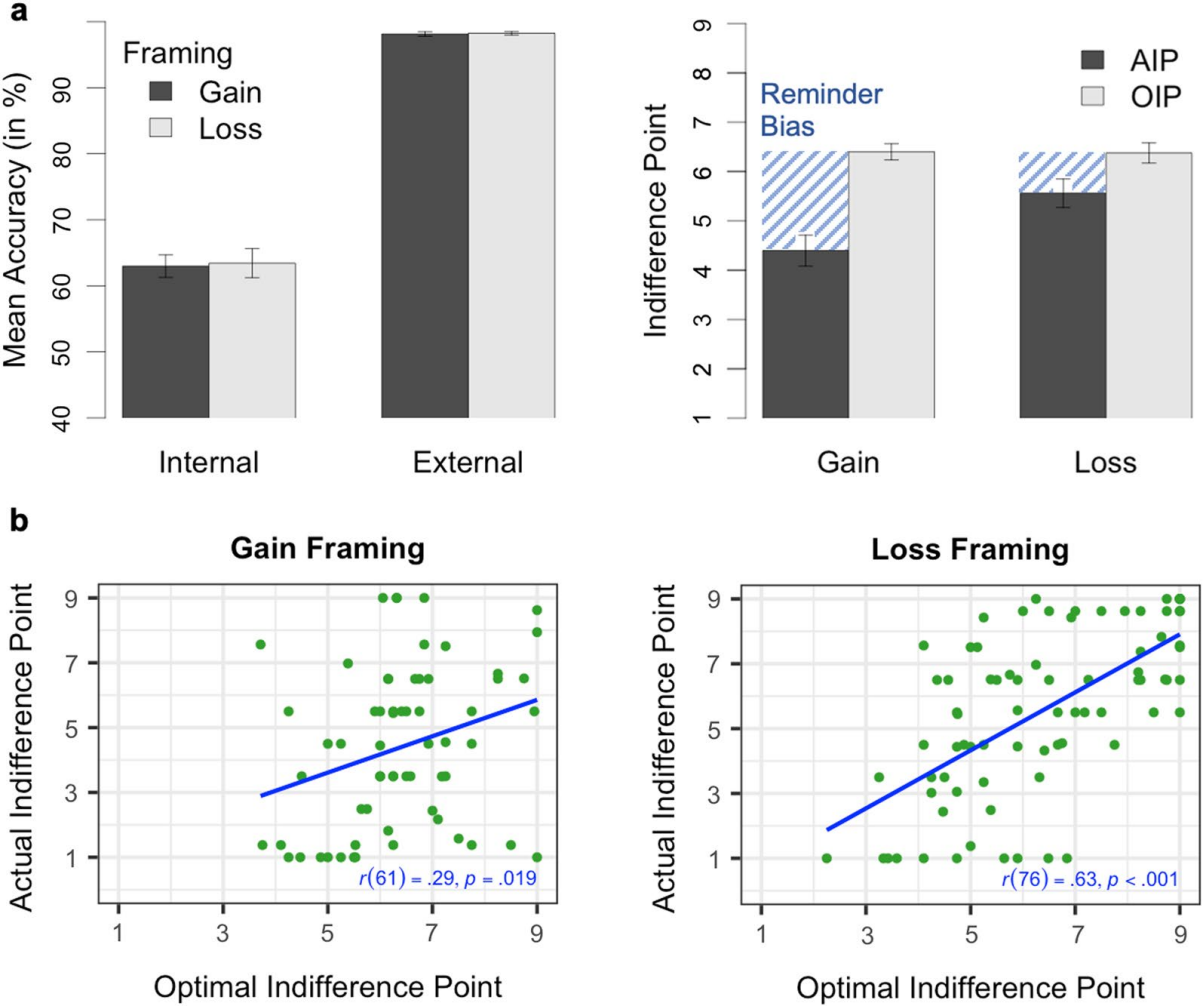
Results on Offloading Behavior from Experiment 1. *Note* **a** In the left panel, mean accuracy for the forced-internal and forced-external trials in the gain and loss framing conditions. Mean optimal (OIPs) and actual indifference points (AIPs) as a function of framing condition in the right panel. The line pattern illustrates the size of the reminder bias defined as OIP minus AIP. **b** Correlation between actual and optimal indifference points for the gain (left panel) and loss (right panel) framing condition. ^a^Error bars represent standard errors

Participants were able to remember almost two-thirds of the special circles using their memory in forced-internal trials, but nearly all of them using reminders in forced-external trials (see Fig. 3a). This data is consistent to the accuracy data found in Gilbert et al. (2020). As an exploratory analysis, we submitted the accuracy data to a 2 (condition: forced-internal vs. forced-external; within) $\times$ 2 (framing: gain vs. loss; between) mixed analysis of variance (ANOVA). Whereas the main effect of condition was significant, *F*(1, 139) = 641.22, *p* < 0.001, η_p_^2^ = 0.82, neither the main effect of framing, *F*(1, 139) = 0.03, *p* = 0.856, η_p_^2^ < 0.01, nor the interaction of framing and condition, *F*(1, 139) = 0.02, *p* = 0.899, η_p_^2^ < 0.01, was significant. Thus, although participants showed a higher task accuracy when using reminders, their overall task performance was not affected by the framing of reminders.

#### Reminder bias

As defined in our preregistration, we investigated the influence of framing on the reminder bias (defined as OIP minus AIP) by submitting the reminder bias scores to a 2 (framing condition: gain vs. loss; between) $\times$ 2 (indifference point: OIP vs. AIP; within) mixed ANOVA (see Fig. 3a). There was a significant main effect of indifference point, *F*(1, 139) = 52.17, *p* < 0.001, η_p_^2^ = 0.27, and a non-significant main effect of framing condition, *F*(1, 139) = 3.45, *p* = 0.065, η_p_^2^ = 0.02. Importantly, the interaction of framing and indifference point was significant, *F*(1, 139) = 10.05, *p* = 0.002, η_p_^2^ = 0.07, with the reminder bias scores, on average, 1.19 points greater in the gain (*M* = 2.00, *SD* = 2.45) compared to loss condition (*M* = 0.81, *SD* = 2.00). We further analyzed whether the reminder bias scores were significantly greater than zero with preregistered one-tailed paired *t* tests (OIPs vs AIPs). Although framing reminders in terms of losses instead of gains resulted in a significant reduction of the reminder bias, the reminder bias was still significant both in the gain, *t*(62) = 6.49, *p* < 0.001, *d*_*z*_ = 0.82, and loss condition, *t*(77) = 3.60, *p* < 0.001, *d*_*z*_ = 0.41. That is, although participants offloaded more optimally under loss framing than under gain framing, participants still offloaded more than optimal also under loss framing.

We ran an exploratory analysis on the relationship between the OIPs and AIPs. To achieve this, we calculated a Pearson’s product–moment correlation on the indifference points for each framing condition. We further tested for the difference between correlations by using the R package cocor (test of significance for independent correlations; Diedenhofen & Musch, 2015). There was a significant positive correlation between the OIPs and AIPs in both conditions (gain: *r* = 0.29, *p* = 0.019; loss: *r* = 0.63, *p* < 0.001; see Fig. 3b), with the correlation being significantly higher in the loss compared to the gain condition, Fisher’s (1925) *z* =  − 2.53, *p* = 0.011. That is, despite participants having a systematic bias toward using reminders, those who derived the most benefit from reminders (i.e., low OIP) were also most likely to use them (i.e., low AIP) and, although evident in both conditions, this relationship was more pronounced when offloading was associated with a loss (see Fig. 3).

## Discussion

Consistent with our hypotheses, we observed a strong reminder bias in the gain condition of Experiment 1. That is, participants offloaded more than optimal, replicating previous findings (Gilbert et al., 2020). In addition, we showed for the first time that the framing of reminders in terms of gains or losses shapes the reminder bias. That is, the reminder bias was largely reduced under loss framing. This is in line with the literature on decision-making (Kahneman & Tversky, 1979), as it suggests that while individuals employ a risk-averse strategy in situations involving gains, they are more inclined to take a risk in situations involving losses. However, it is to note that despite participants being more risk-seeking when facing the prospect of a loss, they still deviated from optimal offloading behavior in the loss framing condition, thus using reminders more than optimal. This finding is consistent with the view that also other factors influence the reminder bias, such as participants’ underconfidence in their memory abilities (Gilbert et al., 2020) and their invested cognitive effort (Sachdeva & Gilbert, 2020). We addressed these points in Experiment 2.

## Experiment 2

In Experiment 2, we again manipulated the framing of reminders as gains or losses, while participants performed the optimal reminders task. In addition, we asked the participants to make metacognitive judgments at the beginning of the experiment. This allowed us to assess their overconfidence or underconfidence in carrying out the task (Gilbert et al., 2020). Further, we rewarded participants depending on their task performance. This acted as a financial incentive to increase both the cognitive effort that participants invest while performing the task and the optimality of their offloading choices (Sachdeva & Gilbert, 2020). Furthermore, we manipulated framing within-participants instead of between-participants in order to account for potential individual differences in framing effects (see Levin et al., 2002). As in Experiment 1, we expected to observe a reminder bias in the gain framing condition, but a reduction (or even reversal) of this bias in the condition where reminders implied a loss. This experiment was preregistered; see https://osf.io/8zvf6/.

### Method

#### Participants

Participants were recruited from the Amazon Mechanical Turk website (http://www.mturk.com), an online marketplace in which participants receive payment for completion of web-based tasks (Crump et al., 2013). Sample size was estimated performing a power analysis with G*Power 3.1 (Faul et al., 2007). The power analysis was based on the meta-analysis of Kühberger (1998), as to our knowledge there was no previous study that has investigated the effect of gain and loss framing within-participants in the context of cognitive offloading.^1^ With a Cohen’s *d* effect size of 0.41 for the within-participants studies in this meta-analysis, at least 49 participants were required for 80% power ($$\alpha$$ = 0.05, two-tailed paired *t* test). This experiment also provided data for a separate unrelated project, which was reported elsewhere (Kirk et al., 2021). Therefore, we were aiming for a higher sample size (i.e., *N* = 300), as this was the intended sample size for the other project. As in earlier studies (Gilbert, 2015a, 2015b), participation was restricted to volunteers aged at least 18 years, located in the USA. We also restricted inclusion to participants with a minimum of 90% Mechanical Turk approval rate. Participation took approximately 45 min, for which participants were guaranteed a base payment of $2, plus an additional bonus of up to $8.67 depending on their task performance. Participants had a mean age of 37.81 years (*SD* = 10.97, *range* = 21–72); 190 reported their gender as male, 108 as female, and 2 as other. Ethical approval was received from the UCL Research Ethics Committee (1584/003) and participants provided informed consent before participating in the study.

#### Optimal reminders task

**Fig. 4:**
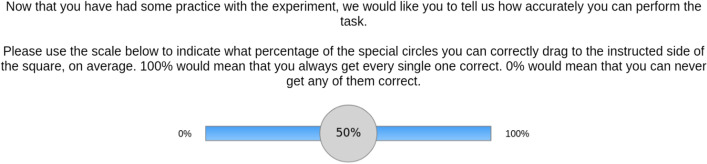
Metacognitive Confidence Judgment. *Note* The metacognitive confidence rating provided us with our metacognitive confidence measure: After participants finished a series of practice trials, they rated their confidence in their ability to accurately remember the delayed intention of dragging the special circles to the respective edges. Participants performed these ratings once (prior to the start of the experiment) separately for the internal and external strategies. For the confidence rating after the forced-external trials, participants received the alternate instruction 'Now that you have practiced doing the task using reminders, we would like you to tell us how accurately you can perform the task when you use this strategy’

We used the same modified version of the optimal reminders task as in Experiment 1, with small adaptations. First, Experiment 2 used an English-speaking MTurk sample, and thus, all items were presented in English. Second, we included metacognitive accuracy judgments in between the practice trials. That is, both after practicing trials on the forced-internal and the forced-external type, participants provided a measure of how confident they were at their ability to perform the task (see Fig. 4). Third, framing was implemented as a within-participants variable. This allowed us to test whether results observed in Experiment 1 can also be observed when comparisons between framing conditions are made within, rather than between individuals. To do this, the task was split into two blocks: gain and loss (with the order of blocks being counterbalanced between participants).

Finally, we did not assign negative values to loss points. That is, while instructions of the gain condition stayed the same, during the loss condition participants received the maximum of points available for this block (100 points per trial) before the beginning of the block. They were presented with the choice between either using their own memory and keeping all their points (losing 0) each time they correctly remembered special circles, or using reminders, and losing points every time they remembered (2–8). Contrasting to Experiment 1, this had the advantage that both conditions were equivalent in terms of the reward participants received, that is the outcomes were phrased to appear as either gains or losses, but objectively they were the same. For example, if offered 7 points to use reminders and a participant chose to use reminders and successfully remembered every special circle, in the gain condition they would earn 70 points. Whereas, in an equivalent trial of the loss condition (i.e., 3 points offered to use reminders), they lost 30 points and thus also retained 70 points. By using this scoring scheme, we were expecting to maximize the comparability of framing conditions, thus reducing the risk of potential data noise. Note that this change in scoring also resulted in Experiment 2 studying the framing effect whereas Experiment 1 investigated the reflection effect (Fagley, 1993).

#### Procedure

Participants first provided informed consent and then completed six practice trials, with the practice trials following the same procedure as in Experiment 1. However, after each pair of consecutive practice trials with and without reminders, participants made their metacognitive judgments reporting how accurately they can perform the task with the respective strategy. Participants were then randomly assigned to the gain or loss condition. In the first experimental block (gain or loss), participants performed a total of 13 (7 free, 6 forced) trials. Due to the within-participants implementation in Experiment 2, participants had to perform trials of both conditions and therefore for practical reasons, that is to keep the task duration within reasonable limits for the participants, we reduced the total trial number per block. As a result of that, the range of the indifference points were changed in accordance with the reduced number of the free-choice trials, that is from 1 to 9, in Experiment 1, to 2 to 8, in Experiment 2. On odd-numbered trials, participants were given a free choice between using internal memory (10 points per special circle) or reminders (2–8 points per special circle, presented in random order). On even-numbered trials, participants alternated between the forced-external and forced-internal trials, with the starting trial type (external or internal) randomized between participants and counterbalanced between gain/loss conditions. Participants then received experimental instructions for the other condition (gain or loss). After finishing the second experimental block (gain or loss; 13 trials as above), participants completed two questionnaires. This was part of the other unrelated project, addressing a different question which is reported in a separate paper (see Kirk et al., 2021, for full details). For a demonstration, the entire experiment can be accessed at.

http://ucl.ac.uk/sam-gilbert/demos/CWPK1/start.html

#### Reward

This time we paid participants depending on their task performance. Paying participants based on their task performance should ensure that they are more motivated to make optimal choices (see Sachdeva & Gilbert, 2020). Implementing this payment allowed us to evaluate whether effects of framing observed in Experiment 1 differ in situations where participants have a financial incentive to choose optimally. To do this, participants were told that they were scoring points, where 300 points were equivalent to $1. They received 600 points at the beginning of the experiment. Then, they were additionally able to earn (or keep) up to 1300 points (i.e., 100 points per trial) in each half of the experimental trials. Therefore, the earnings could range between 600 points ($2) and 3200 points ($10.67). The experiment was advertised as having a base payment of $2, which participants received simply for taking part, with the additional earnings sent to participants afterward as a bonus payment.

#### Design

We entered framing (gain vs. loss) as within-participants variable into our design. The variables of interest were the same as in Experiment 1, with the indifference points bounded to the range 2 to 8. In addition, measures regarding the metacognitive judgments were added:

- Internal metacognitive confidence. This is the response made to the metacognitive accuracy prediction following practice trials using internal memory (see Fig. 4).
- External metacognitive confidence. This is the response made to the metacognitive accuracy prediction following practice trials using reminders.
- Internal metacognitive bias. This is the difference between metacognitive confidence and actual accuracy on forced-internal trials. A positive number would indicate overconfidence of their own memory abilities.
- External metacognitive bias. This is the difference between metacognitive confidence and actual accuracy on forced-external trials. A positive number would indicate overconfidence of their performance when using reminders.

#### Exclusion criteria

Similar to Experiment 1, participants were excluded if (a) their accuracy in the forced-internal condition was lower than 10%, averaged across the gain and loss conditions; (b) accuracy in the forced-external condition was lower than 70%, averaged across the gain and loss conditions; (c) accuracy on the forced-internal trials was higher than forced-external trials in either condition; (d) there was a negative point biserial correlation between points offered for correct responses on each trial using reminders (2–8) and choice of strategy (0 = own memory, 1 = reminders; this excludes participants who were more likely to set reminders when it earned them fewer points, suggesting random strategy selection); (e) reminder bias score (averaged across the gain and loss conditions) exceeded 3 median absolute deviation units (MAD; Leys et al., 2013); (f) difference in reminder bias scores between the two conditions exceeded 3 MAD units; and (g) internal metacognitive bias score exceeded 3 MAD units. Data collection continued until the study had the appropriate power (*N* = 300) following exclusion (64 excluded). The data are publicly available (https://osf.io/8zvf6/).

### Results

#### Accuracy

**Fig. 5:**
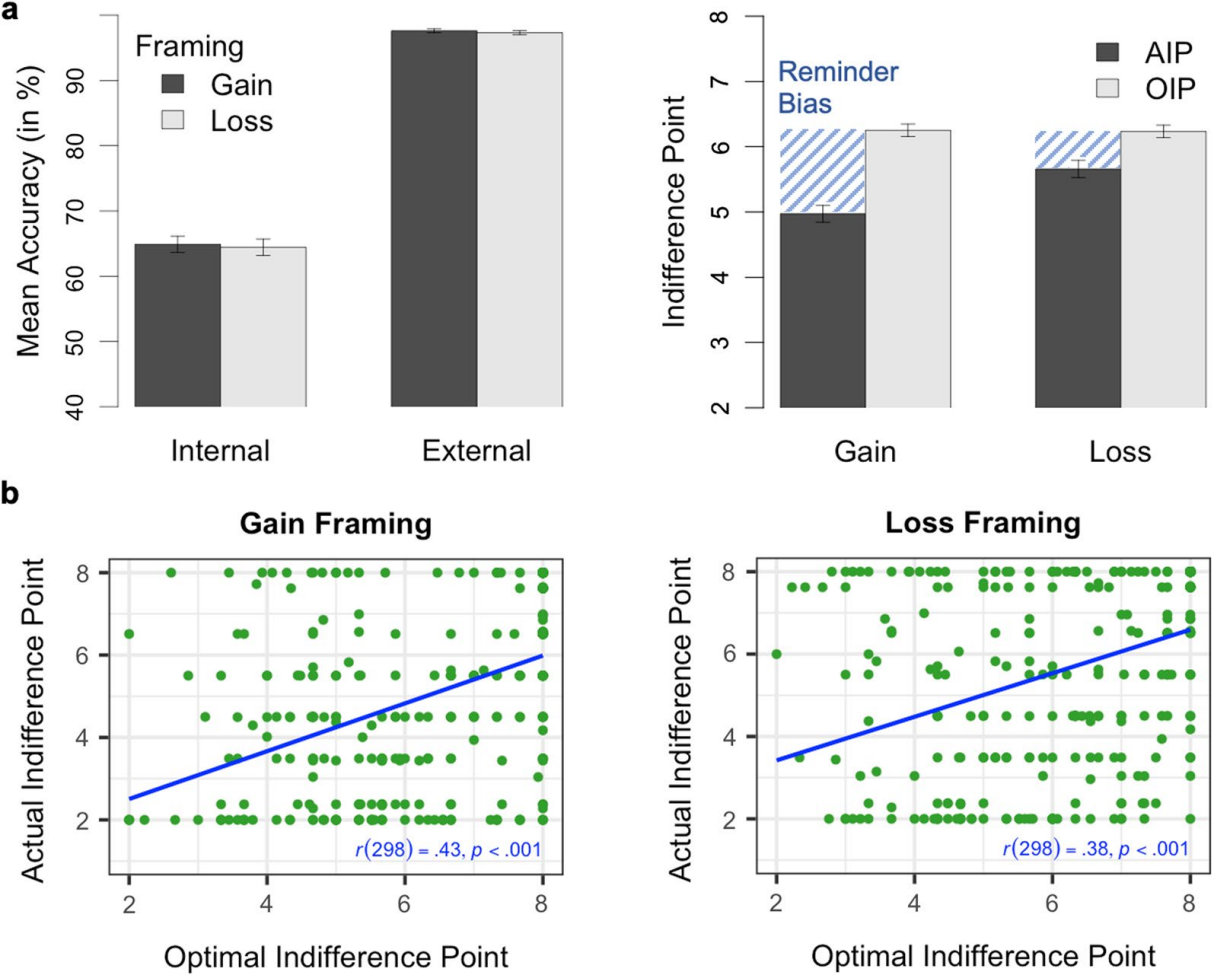
Results on Offloading Behavior from Experiment 2. *Note* **a** In the left panel, mean accuracy for the forced-internal and forced-external trials in the gain and loss framing condition. Mean optimal (OIPs) and actual indifference points (AIPs) as a function of framing condition in the right panel. The line pattern demonstrates the size of the reminder bias defined as OIP minus AIP. **b** Correlation between actual and optimal indifference points for the gain and loss framing condition. ^a^Error bars represent standard errors

Participants remembered almost two-thirds of the special circles using their memory in forced-internal trials, and they remembered nearly all of the special circles when using reminders in forced-external trials (see Fig. 5a).^2^ This replicates the accuracy data found in Experiment 1 and previous research (Gilbert et al., 2020). We submitted the accuracy data to a 2 (condition: forced-internal vs. forced-external; within) $\times$ 2 (framing: gain vs. loss; within) ANOVA with repeated measures on both variables. We again obtained a significant main effect of condition, *F*(1, 299) = 991.36, *p* < 0.001, η_p_^2^ = 0.77, but no significant main effect of framing, *F*(1, 299) = 0.33, *p* = 0.565, η_p_^2^ < 0.01, and a non-significant interaction of framing and condition, *F*(1, 299) = 0.02, *p* = 0.892, η_p_^2^ < 0.01. This indicates that accuracy was significantly affected by the use of reminders, but not by the framing of reminders, just as in Experiment 1.

#### Reminder bias

Our key hypotheses were tested using the reminder bias scores. As preregistered, we analyzed the reminder bias scores (OIP minus AIP) with two-tailed one-sample *t *tests. We observed a significant reminder bias both in the gain framing condition, *t*(299) = 10.32, *p* < 0.001, *d* = 0.60, and in the loss framing condition, *t*(299) = 4.37, *p* < 0.001, *d* = 0.25. To evaluate whether these reminder bias scores differed as a function of framing conditions, we performed a preregistered two-tailed paired *t* test comparing reminder bias scores of the gain and loss conditions. This analysis yielded a reminder bias that was, on average, 0.70 points greater in the gain condition (*M* = 1.28, *SD* = 2.14) compared to the loss condition (*M* = 0.58, *SD* = 2.28), *t*(299) = 5.05, *p* < 0.001, *d*_*z*_ = 0.29.

Given that we manipulated framing within-participants in Experiment 2, we obtained two separate reminder bias scores for each participant, once for the gain condition and once for the loss condition. We calculated a preregistered Pearson’s product–moment correlation on these two scores to check whether individual differences in task behavior were significantly related across framing conditions. We found that the reminder bias scores in the two framing conditions were significantly correlated, *r* = 0.41, *p* < 0.001. This indicates that low (high) reminder bias scores in one framing condition were associated with low (high) scores in the other condition. As in Experiment 1, we exploratorily performed a Pearson’s product–moment correlation between the OIPs and AIPs for both framing conditions (see Fig. 5b). There was a significant correlation both under gain framing, *r* = 0.43, *p* < 0.001, and loss framing, *r* = 0.38, *p* < 0.001, with the difference between correlations being non-significant, Pearson and Filon’s (1898)* z* = 0.83, *p* = 0.405. (Note that Experiments 1 and 2 differ in this respect as this time we tested for the difference between dependent, non-overlapping correlations using the R package cocor; Diedenhofen & Musch, 2015.) Similar to Experiment 1, participants who benefited the most from using reminders (i.e., low OIP) were more likely to offload intentions (i.e., low AIP). However, in contrast to Experiment 1, this relationship between actual task performance and offloading behavior did not differ between framing conditions.

#### Metacognitive bias

**Fig. 6:**
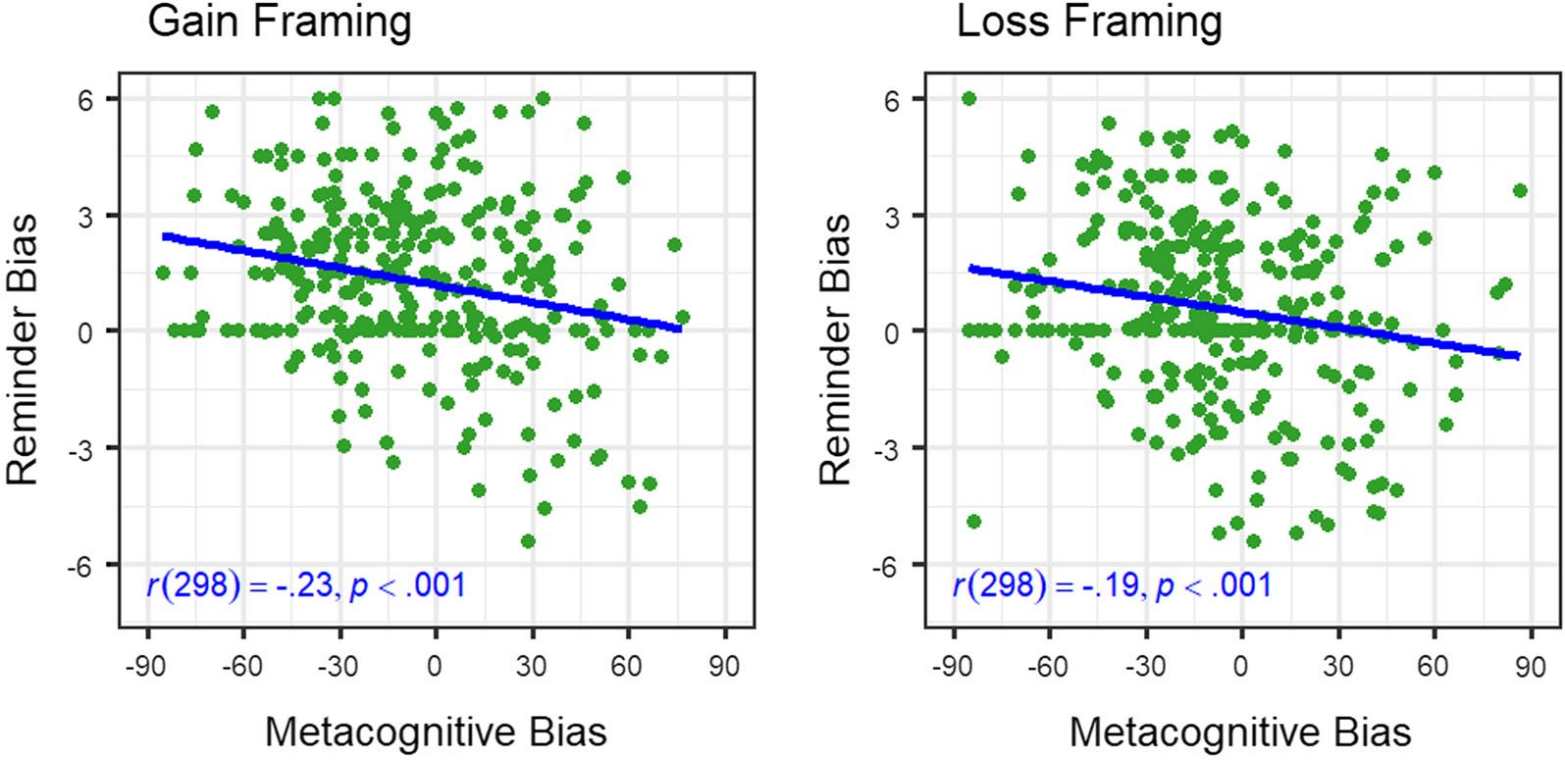
Relationship between Metacognitive Confidence and Offloading Behavior. *Note* Correlation between individuals’ metacognitive bias and reminder bias scores, separately for both framing conditions. Negative metacognitive bias scores illustrate underconfidence in memory ability and positive reminder bias scores represent overuse of reminders

As reported in the preregistration, we performed three additional analyses involving metacognitive judgments. First, we performed separate two-tailed one-sample *t* tests on the internal and external metacognitive bias scores against zero to test whether participants were under- or overconfident using the two strategies. Both the internal, *t*(299) =  − 3.65, *p* < 0.001, *d* = 0.21, and external, *t*(299) =  − 12.96, *p* < 0.001, *d* = 0.75, metacognitive bias scores were significant (*M* =  − 6.69, *SD* = 31.76; and *M* =  − 11.21, *SD* = 14.97, respectively). This indicated that participants were significantly underconfident about their memory abilities and their accuracy using reminders. Second, we assessed whether participants’ underconfidence in their internal judgments was associated with a higher reminder bias by calculating a Pearson’s product–moment correlation between the internal metacognitive bias score and the reminder bias score for both framing conditions (see Fig. 6).^3^ A significant negative correlation was found in both framing conditions between reminder bias and internal metacognitive judgments (gain: *r* =  − 0.23, *p* < 0.001; loss: *r* =  − 0.19, *p* < 0.001), indicating that underconfident participants were more biased toward reminders, regardless of framing condition in free-choice trials. Third, we assessed whether the relationship between reminder bias and internal metacognitive bias differed as a function of framing condition by using the R package cocor (test for dependent, non-overlapping correlations; Diedenhofen & Musch, 2015). We found no evidence that framing altered the relationship between internal confidence and the tendency to use reminders, Fisher’s (1925) *z* =  − 0.62, *p* = 0.537.

In an exploratory analysis, we further investigated whether framing influences the reminder bias over and above participants’ internal metacognitive bias. To achieve this, we fitted mixed-effects models (R package lme4; Bates et al., 2015) on the reminder bias scores with random intercepts for the participant effect. To initiate the model selection process, we compared the intercept-only model (Akaike information criterion [AIC]: 2630.50) with a model with internal metacognitive bias as continuous fixed factor (AIC: 2623.20). We found significant differences between the models in a likelihood-ratio test, $\chi$ ^2^(1) = 9.29, *p* = 0.002, indicating that the reminder bias was significantly increased by participants’ underconfidence in their memory abilities $.$ We thus selected the model with the one fixed effect and compared it with a model which also included the framing (gain vs. loss) as another fixed factor (AIC: 2601.70). Again, a likelihood-ratio test revealed significant differences, $\chi$ ^2^(1) = 23.51, *p* < 0.001. We thus selected the model with the two fixed factors and compared it with a model including the interaction of internal metacognitive bias and framing as the third fixed effect (AIC: 2603.60), with no significant differences between the models in a likelihood-ratio test, $\chi$ ^2^(1) = 0.09, *p* = 0.761. Thus, we retained the simpler model (additive terms only). This suggests that framing adds an additive predictive value to the reminder bias, that is beyond the effect of participants’ metacognitive miscalibration of their internal memory abilities.

### Discussion

Consistent with our study’s key hypotheses, Experiment 2 illustrates that while participants choose to use reminders more than would be optimal, this systematic bias is reduced by the framing of reminders in terms of losses. This indicates that, despite receiving financial incentive to offload optimally, individuals employ a risk-averse approach in situation involving gains but a more risk-seeking strategy when offloading implies a loss (see Kahneman & Tversky, 1979). Furthermore, in the scope of participants’ metacognitive confidence ratings, Experiment 2 demonstrates that irrespective of the framing of reminders individuals are underconfident about their memory abilities and that this erroneous metacognitive underconfidence in turn is associated with a greater bias toward using reminders in both framing scenarios, which replicates the negative relationship between internal metacognitive bias and reminder bias found in Gilbert et al. (2020). Further, we show that the framing of reminders influences participants choice in using reminders over and above participants’ internal metacognitive bias.

## General discussion

The present research aimed at achieving more optimal decisions in offloading situations, by assessing whether the framing of reminders (Kahneman & Tversky, 1979) influences how optimally intentions are offloaded onto external devices. In two experiments, we manipulated the framing of reminders in terms of gains or losses, once between-participants (Experiment 1) and once within-participants (Experiment 2). Experiment 2 further measured metacognitive judgments and provided participants with financial incentives to offload optimally. Participants were less biased toward using reminders when reminders were framed as losses instead of gains. This was also true when participants received financial incentives for optimal offloading in Experiment 2. Further, the bias toward using reminders was higher the more underconfident participants were about their internal memory abilities, irrespective of the framing of reminders. The framing of reminders as well as participants’ internal metacognitive bias were two independent factors, each providing unique predictive value regarding the optimality of participants’ offloading choices.

In line with previous literature on decision-making (Kahneman & Tversky, 1979), we observed that in situations involving gains, individuals employed a risk-averse offloading strategy in situations involving gains and as a result used more reminders, compared to situations where offloading implied a loss. The experiments’ findings are thus consistent to studies demonstrating framing effects and reflection effects on decision-making in other domains (e.g., Bless et al., 1998; Highhouse & Paese, 1996; Mather et al., 2012; for a review see Baucells & Villaís, 2010; Kühberger, 1998; Piñon & Gambara, 2005; Steiger & Kühberger, 2018; Tversky & Kahneman, 1981). However, our study is the first to demonstrate effects of gain and loss framing in the context of cognitive offloading.

A possible explanation for the differences in offloading strategies between framing conditions concerns the certainty effect (Kahneman & Tversky, 1979). As demonstrated in both experiments, participants in the gain condition were systematically biased toward using reminders, thus preferring the option that provided more certainty regarding the outcome. This indicates that when the choice between reminders or internal memory is framed in terms of gains, individuals are risk-averse and prefer the certain over the risky outcome, which is in line with the certainty effect (Kahneman & Tversky, 1979). In the loss condition, however, the systematic overuse of reminders was reduced indicating that participants were more willing to take the risky outcome of potentially forgetting a delayed intention and therefore losing a greater number of points by using their internal memory. This finding is consistent to the literature on decision-making (Kahneman & Tversky, 1979), as it demonstrates that a switch from gain to loss framing also causes a switch from a risk-averse strategy to a more risk-seeking strategy. Moreover, our study illustrates that this switch in strategy also applies to cognitive offloading.

However, even though participants were more risk-seeking in situations involving losses, in both experiments the reminder bias was still present in the loss framing condition. Therefore, participants were still risk-averse and preferred to engage in offloading behavior even though reminders implied a loss. That is, despite reminders being framed in terms of losses participants still preferred the outcome that was more certain (using reminders) over the uncertain and more variable outcome (using internal memory). Hence, we observed a preference shift but no preference reversal. This suggests that, in the context of cognitive offloading, the framing of choices in terms of losses does not loom large enough to reverse the systematic preference for certain outcomes, thus leaving some amount of reminder bias. This finding may be due to domain-related characteristics of offloading situations which, by nature, could have impeded the reversal of risk preferences between framing conditions (for a review on moderating variables under gain and loss framing, see Piñon & Gambara, 2005). For example, individuals are usually more sensitive for decisions under risk, rather than uncertainty (Tversky & Fox, 1995). Thus, adding accuracy-based feedback for each individual, that is ensuring that the probabilities associated with the respective strategies are known, may dissolve, at least, some domain-specific constraints of offloading situations (which one could argue would then lead to a further reduction of the reminder bias).

A second explanation for individuals not being entirely risk-seeking in the loss framing condition concerns other influence factors that were still present in situations involving losses, providing a separate effect on the decision to use reminders. Specifically, participants’ internal metacognitive bias is one such additional factor influencing participants’ offloading decisions (Boldt & Gilbert, 2019; Engeler & Gilbert, 2020). In Experiment 2, we measured participants’ metacognitive judgments and we observed that our participants were underconfident in their internal memory capabilities and that this internal metacognitive bias also contributed to the reminder bias, irrespective of the framing of reminders. Interestingly, Engeler and Gilbert (2020) indicate that correcting participants’ internal metacognitive judgments via positive feedback about their actual task performance may not be sufficient to eliminate the reminder bias. That is, even though participants no longer needed to be underconfident in their memory abilities, they still showed a systematic bias toward using reminders (Engeler & Gilbert, 2020). If one combines those findings with the results of our Experiment 2, which suggested that the framing of reminders influenced the reminder bias over and above participants’ internal metacognitive bias, one could argue that the framing of reminders and participants’ internal metacognitive bias are two independent and additive factors influencing participants offloading behavior.

A third influence factor on the choice of using reminders is cognitive effort. When participants are provided with a performance-based financial incentive, they invest more cognitive effort resulting in an increased overall task performance and a more optimal use of reminders (Sachdeva & Gilbert, 2020). Despite providing a performance-based financial incentive in our second experiment, participants still showed a significant (though reduced) reminder bias when reminders were framed in terms of losses. The combination of a performance-based financial incentive with a loss framing was not sufficient to eliminate the reminder bias. This is consistent to the view that some decisions are rather difficult to debias, especially if relying on a nonlinear relationship between the decision prospects (gain/benefit vs. loss/cost) and the subjective values assigned to those prospects (Arkes, 1991). Future research should thus continue investigating the joint––and hopefully debiasing––role of framing, metacognition, and cognitive effort on cognitive offloading, with a specific focus on how to best achieve more optimal decisions in offloading situations.

Despite the overall reduction of the reminder bias induced by loss framing, we also observed a rather consistent tendency of overusing reminders across participants. In particular, the correlation analysis between individual reminder biases in the gain framing and loss framing conditions in Experiment 2 indicated that individual differences in participants’ offloading strategies were consistent over the two framing conditions. This may suggest that participants tendency to overuse reminders may be partially due to a preference for consistency in performance, regardless of choice. That is, by having a general tendency to use reminders, participants are consistent and avoid the higher variability in accuracy that occurs when using an internal memory strategy. Therefore, participants may be more willing to avoid variance in performance even when using their internal memory may result in more correct responses and a higher financial reward. This explanation is consistent with research proposing that individual’s preferences are risk-averse in terms of mental effort, opting for a fixed amount of effort rather than a variable amount (Apps et al., 2015). This preference may be more prevalent when the task demands are difficult, especially when one choice may require significantly more cognitive effort expenditure, as in the current study.

Consistent with research on the original optimal reminders task (Gilbert et al., 2020), participants were consistently biased toward using reminders in the gain condition of both experiments, thus engaging in offloading behavior more than optimal (Scarampi & Gilbert, 2020). However, the reminder bias was still evident in the loss condition. With this first experimental demonstration that individuals deviate from optimal decision-making in situations where offloading involves possible losses, we add to previous literature. Furthermore, as both experiments varied with respect to multiple factors (e.g., different samples; manipulation within-participants vs. between-participants; different scoring schemes; see above, for further details), we demonstrate that the systematic overuse of reminders constitutes a fundamental bias in human decision-making that occurs independently of subtle variations in context (De Martino et al., 2006; Hartley & Phelps, 2012; Mulder et al., 2012; Tom et al., 2007).

## Implications

Offloading information onto external artifacts can significantly help to fulfil future intentions, with participants consistently remembering more than 90% of intentions using reminders in our experiments. This benefit of offloading on memory performance replicates results found in previous studies (Engeler & Gilbert, 2020; Gilbert et al., 2020; Scarampi & Gilbert, 2020). However, using external devices in daily life does not always incur a gain. For example, although offloading can help remember intentions when memory resources become taxed, it takes time and effort to set them up. Even though these individual costs of setting a reminder may be small, they mount to an unacceptable level if reminders were used for every one of the dozens, maybe hundreds, of things one intends to do in an ordinary day. Other costs of using reminders may only become apparent in the long run. That is, apart from needing more time and effort, individuals miss out important cognitive exercise when setting up a reminder, possibly affecting cognitive functioning eventually (see Grinschgl et al., 2021b). In fact, the human brain is highly plastic (Green & Bavelier, 2008), requiring exercise to establish and maintain proper functioning, especially at an older age (Buckner, 2004; Joubert & Chainay, 2018; Morrison & Chein, 2011; Park & Bischof, 2013; Small, 2001). Therefore, when aiming toward an optimal offloading behavior, in real life one faces the challenge how to deal with such costs and benefits of using reminders (i.e., how harmful might it be to use the offloading strategy instead of training the brain and how does this relate to the resulting performance).

In our experiments, we demonstrated that while individuals were biased toward using reminders when facing the prospect of a gain, individuals’ offloading decisions tended to be more optimal in situations involving losses. Therefore, framing in the sense of highlighting either costs or benefits of offloading may also be a promising tool in achieving a more optimal offloading regarding the relation of costs and benefits in daily life. Specifically, with using framing as a tool one may be able to develop interventions targeting the perspective individuals adopt in offloading contexts. That is, in situations where people are biased one way or the other (toward or away from offloading), the framing of the instructions could be used to make peoples’ offloading decisions more optimal and this approach could be used to create personalized interventions based on each individual’s particular bias. For example, directing individuals’ focus on the costs that each strategy (using reminders or memory) has for their daily life functioning may encourage them toward more flexible adaptions of their offloading strategy. In a clinical context, for example, this may be a promising technique in establishing an optimal balance between functional and compensational rehabilitation for patients with neurological disease (see Thöne-Otto & Walther, 2008, for further details).

## Conclusion

With the present research, we argue that the optimality with which individuals engage in offloading behavior can be shaped by the way offloading situations are framed. When situations are framed in terms of gains, reminders are preferred, and when situations are framed in terms of losses, individuals are less risk-averse and use reminders more optimally. Whereas previous attempts in achieving more optimal decisions in offloading situations have been following a mainly metacognitive approach (Engeler & Gilbert, 2020; Gilbert et al., 2020; Risko & Gilbert, 2016), the present study adds the concept of framing to this research area. We demonstrate that individuals’ offloading strategies under gain and loss framing are affected in line with the risk preferences predicted from previous literature on decision-making (Kahneman & Tversky, 1979), leading to more optimal decisions when emphasizing the costs (losses) rather than the benefits (gains) of offloading behavior. Thus, we claim that it is essential to move forward toward an inclusive theory of offloading behavior, to expand our knowledge on how to best improve humans’ decision-making in offloading contexts.

## Data Availability

The datasets generated and analyzed during the current study as well as the materials are available in the OSF repository, https://osf.io/8shkf/ (Experiment 1), https://osf.io/8zvf6/ (Experiment 2).

## Footnotes

1 At this point, we want to clarify that both experiments were conducted independently from two separate working groups. We follow the same guidelines of transparency and openness as in Experiment 1; however, some parts of the methodical and statistical approach differ between experiments. Also, note that in the original order Experiment 2 has been performed prior to Experiment 1. We changed experiments within the scope of the current paper as we perceived this order to be more coherent.

2 Please note that Kirk et al. (2021) used the data of the gain condition for their analyses. Therefore, some values concerning offloading behavior in the gain condition are identical between the two projects.

3 Note that according to our preregistration we planned to correlate the internal metacognitive bias score with the reminder bias within each condition (gain or loss). However, we realized that this could yield spurious self-correlations due to non-independent observations, because the forced-internal accuracy score was used to calculate both the internal metacognitive bias and the reminder bias. To avoid this potential statistical artifact, we deviated from the preregistration by calculating the internal metacognitive bias from the opposite experimental condition (i.e., the reminder bias in the gain condition was correlated with the internal metacognitive bias from the loss condition, and vice versa). This ensured that the correlation analyses met the assumption of independent observations. Results were similar when the analyses were conducted as specified in the original preregistration.
